# Constraints of Access to Agricultural Information in Africa: A Systematic Review

**DOI:** 10.1155/tswj/4980057

**Published:** 2025-12-11

**Authors:** Melese Abebaw Abate

**Affiliations:** ^1^ Department of Rural Development and Agricultural Extension, Mekdela Amba university, Tuluawliya, Amhara Region, Ethiopia, mkau.edu.et

**Keywords:** access, Africa, agriculture, constraints, information, source

## Abstract

This review is aimed at assessing the constraints of access to agricultural information in African countries. To conduct this, secondary data from articles were used. A multistage sampling procedure was used to select Ethiopia, Cameroon, Ghana, Nigeria, South Africa, Sudan, and Uganda as the sample countries for the review. The collected data was combined and interpreted for a general conclusion and recommendation. The review highlights that in Africa, agricultural information was accessed from radio, television, mobile phones, computer and internet, face‐to‐face contact with extension workers, fellow farmers, input suppliers, town criers, agricultural research centers, and printed materials such as posters, magazines, newspapers, school/college notes and books, manuals, billboards, and bulletins. However, farmers face several challenges to access the information, such as illiteracy; limited information sharing among farmers; religious beliefs; lack of cooperative membership; unavailability or inappropriateness of information sources; the absence of aids to present the information; the failure to use local language; complex information; high costs of both information and inputs; inadequate or unqualified extension workers; a lack of farmer training, workshops, and seminars; insufficient reading materials; low extension–farmer linkages; poor public relations by extension workers; the long distance of training centers from farmers′ homes; lack of rural electrification; and the absence of rural networks. There should be an expansion of information and communication technology for transferring agricultural information to the farmers in African countries.

## 1. Introduction

Agriculture serves as a primary driver of economic growth, providing food, employment, and foreign exchange in most developing countries [1, 2]. The agricultural economy employs 65%–70% of Africa′s labor force and typically accounts for 30%–40% of GDP. It provides a livelihood for 75% of the people who live in rural areas. It also provides raw materials for industries, such as textiles, food processing, and biofuels, and is a major source of export earnings, particularly for cash crops like cocoa, coffee, tea, and cotton, to facilitate international trade.

While agriculture is the primary livelihood for rural households in Africa, it does not effectively contribute to reducing rural poverty or ensuring food security. The inefficiency of agriculture was caused by limited access to agricultural information for the farmers [3]. Lack of climate‐related information by the rural households affects the performance of agriculture [4]. As the demand for agricultural production rises, land available for farming remains limited, and population growth continues in African countries, it is crucial to have up‐to‐date information to address and mitigate the various constraints of agricultural productivity [5, 6].

Information on modern farming methods on weather forecasts; planting of agricultural products; crop and livestock management, including pest and weed control; harvesting; and postharvest storage techniques can significantly improve the efficiency and effectiveness of farming. Access to agricultural information was used to upgrade the knowledge of the farmers about new technologies and increase their probability of adopting the practice in farming [7]. It was important for the overall development of agriculture [8] and for the eradication of rural poverty [9]. There is no doubt that information is important from planning up to the production stage of farming [10]. Specifically, agricultural information accessed from radio and training was important for giving awareness about the avoidance of bush burning and the use of organic and inorganic fertilizers to increase the crop yield [11].

Several studies have been carried out on the barriers to accessing agricultural information in various African countries. For instance, in Botswana, Ghana, Kenya, and Uganda, access to ICT to improve the agricultural sector was influenced by low capacity and inadequate infrastructure facilities [12]. The major constraints to the use of various information sources were poor marketing facilities, lack of credit, and poor infrastructures, as well as poor extension services [8]. These and other findings reveal that constraints vary from one country to another, even from district to district in the same country. There is no published comprehensive report on sources of agricultural information and constraints of access to agricultural information in Africa. To fill this, it is important to conduct a systematic review on constraints to accessing agricultural information in African countries.

## 2. Methods

### 2.1. Inclusion and Exclusion Criteria

Articles focused on constraints of access to agricultural information in selected sample countries and written in english were the sample Literatures of the review. Books and newspapers were not used as the sample for this review (Table 1).

**Table 1:** Inclusion and exclusion criteria.

**No**	**Inclusion criteria**	**Exclusion criteria**
1	Focused on constraints of access to agricultural information	Focused on another topic
2	Conducted in Ethiopia, Uganda, Ghana, Nigeria, Cameroon, Sudan, and South Africa	Conducted in other countries
2	Written in the English language	Written in another language
5	Articles	Books and newspapers

*Note:* own estimation, 2025.

### 2.2. Sampling Procedure

**Figure 1:**
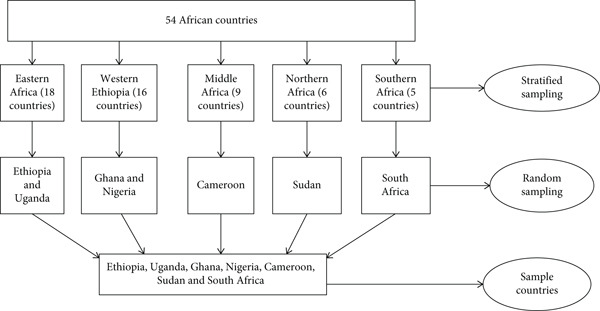
Sampling procedure. Source: own computation, 2025.

Multi‐stage sampling procedure was used to select the sample countries. First, African countries were stratified into five regions as Western, Eastern, Middle, Northern, and Southern Africa. Sixteen countries are located in Western Africa, and the other eighteen countries are found in Eastern Africa, while nine, six, and five countries are located in Middle, Northern, and Southern Africa, respectively. Next, due to a higher number of countries in Western and Eastern Africa, two countries, Ghana and Nigeria from Western Africa and Ethiopia and Uganda from Eastern Africa, were selected by using a random sampling technique from each region. Additionally, Cameroon, Sudan, and South Africa were chosen from Middle, Northern, and Southern Africa, respectively, by using a random sampling technique from each region (Figure 1).

### 2.3. Types of sample literature

Thirty‐five research articles were the sample literature to assess constraints of access to agricultural information in Africa (Table 2).

**Table 2:** Types of sample literature.

**No.**	**Name of the author**	**Type of document**	**Year of publishing**	**Conducted in**
1	Ndemdou and Fongang	Article	2021	Cameroon
2	Guillaume et al.	Article	2019	Cameroon
3	Megerssa et al.	Article	2020	Ethiopia
4	Tadesse	Article	2008	Ethiopia
5	Gebru Brhane et al.	Article	2017	Ethiopia
6	Kelemu	Article	2017	Ethiopia
7	Tegegne and Alemu	Article	2019	Ethiopia
8	Yimer	Article	2015	Ethiopia
9	Adjoe et al.	Article	2010	Ghana
10	Amuda and Thompson	Article	2010	Ghana
11	Tham‐Agyekum et al.	Article	2024	Ghana
12	Aidoo and Freeman	Article	2016	Ghana
13	Lamptey et al.	Article	2016	Ghana
14	Codjoe et al.	Article	2013	Ghana
15	Awuku Manteaw	Article	2022	Ghana
16	Obeng‐Koranteng et al.	Article	2017	Ghana
17	Obidike	Article	2011	Nigeria
18	Amidu et al.	Article	2021	Nigeria
19	Ogbonna and Anunobi	Article	2022	Nigeria
20	Achichi et al.	Article	2023	Nigeria
21	Ogboma	Article	2010	Nigeria
22	Adio et al.	Article	2016	Nigeria
23	Oladele	Article	2015	South Africa
24	Mdoda et al.	Article	2024	South Africa
25	Popoola et al.	Article	2020	South Africa
26	Adam Elradi et al.	Article	2021	Sudan
27	Musa et al.	Article	2013	Sudan
28	Yahia and Bello	Article	2018	Sudan
29	Hamad et al.	Article	2018	Sudan
30	Freeman and Qin	Article	2020	Uganda
31	Mubangizi et al.	Article	2004	Uganda
32	Van Campenhout et al.	Article	2021	Uganda
33	Mubangizi et al.	Article	2018	Uganda
34	Abdul‐Salam and Phimister	Article	2017	Uganda
35	Masuki et al.	Article	2010	Uganda

*Note:* literature review, 2025.

## 3. Methods of Data Collection and Interpretation

First, the reviewer searches and downloads articles which focused on constraints of access to agricultural information and related topics conducted in seven sample countries. Second, the reviewer reads and understands the findings of all of the sample literatures. Third, the findings are organized, edited, and compiled. Fourth, the reviewer generalizes and interprets the findings for discussion and explanation.

## 4. Results and Discussion

### 4.1. Sources of Agricultural Information for the Rural Households in Africa

Radio, television, mobile phones, agricultural extension workers, and colleague farmers were the primary and most influential sources of agricultural information for rural households in most African countries. Moreover, health extension workers, input suppliers, seed/grain stockists, workshop/seminar, town criers, billboard, and bulletins were also used as sources of agricultural information (Table 3). Not all sources were utilized for accessing all agricultural information; instead, the choice of sources was based on the particular circumstances of each country.

**Table 3:** Sources of agricultural information.

**No**	**Sources**	**Country**	**Evidence**
1	Radio	Cameroon, Ethiopia, Ghana, Nigeria, Sudan, and Uganda	Guillaume et al. [13], Megerssa et al. [14], Gebru Brhane et al. [6], Kelemu [15], Yimer [16], Adjoe et al. [17], Amuda and Thompson [11], Tham‐Agyekum et al. [18], Awuku Manteaw [19], Obeng‐Koranteng et al. [20], Achichi et al. [21], Adio et al. [22], Musa et al. [23], Yahia and Bello [24], Freeman and Qin [25], Mubangizi et al. [26], Mubangizi et al. [27], Abdul‐Salam and Phimister [28], Popoola et al. [4]
2	Television	Ethiopia, Ghana, Nigeria, Sudan, and Uganda	Megerssa et al. [14], Kelemu [15], Yimer [16], Adjoe et al. [17], Amuda and Thompson [11], Tham‐Agyekum et al. [18], Awuku Manteaw [19], Achichi et al. [21], Adio et al. [22], Musa et al. [23], Yahia and Bello (2018), Freeman and Qin [25], Abdul‐Salam and Phimister [28], Popoola et al. [4]
3	Mobile, computer, and Internet	Cameroon, Ethiopia, Ghana, Nigeria, South Africa, Sudan, and Uganda	Ndemdou and Fongang [29], Gebru Brhane et al. [6], Adjoe et al. [17], Lamptey et al. [30], Tham‐Agyekum et al. [18], Adio et al. [22], Mdoda et al. [31], Adam Elradi et al. [32], Musa et al. [23], Hamad et al. [33], Freeman and Qin [25], Abdul‐Salam and Phimister [28], Popoola et al. [4]
4	Extension agent (through face‐to‐face contact)	Ethiopia, Ghana, Nigeria, South Africa, and Uganda	Tadesse [34], Gebru Brhane et al. [6], Kelemu [15], Awuku Manteaw [19], Obidike [35], Amidu et al. [36], Ogboma [37], Oladele [38], Mubangizi et al. [26], Mubangizi et al. [27], Popoola et al. [4], Codjoe et al. [39]
5	Colleague farmers, input suppliers, and town criers	Ethiopia, Ghana, Nigeria, and Uganda	Gebru Brhane et al. [6], Aidoo and Freeman [40], Awuku Manteaw [19], Obeng‐Koranteng et al. [20], Ogbonna and Anunobi [41], Achichi et al. [21], Ogboma [37], Adio et al. [22], Mubangizi et al. [27], Popoola et al. [4]
6	Agricultural research center	Ethiopia and Uganda	Gebru Brhane et al. [6], Kelemu [15], Mubangizi et al. [26]
7	Printed materials, including posters, magazines, newspapers, school/college notes and books, manuals, billboards, and bulletins	Nigeria, Sudan, and Uganda	Ogboma [37], Obidike [35], Musa et al. [23], Mubangizi et al. [26]

*Note:* literature review, 2025.

Radio has long been a key tool in rural areas due to its wide reach, affordability, and accessibility. In many African regions, radio broadcasts are used to transfer agricultural information to the farmers. Radio programs often provide localized content, allowing farmers to receive information tailored to their specific environments and challenges. With the rise of community radio stations, information is also shared in local languages, making it more accessible to farmers who may not speak official national languages. It is used to access agricultural advisory services to improve the sector [13]. It is the most frequent source of information on fertilizer application, improved crops, and credit facilities [11], and it is used as the source of information about the application of fertilizer and weedicides to increase tiger nut (*Cyperus esculentus*) production [20].

Television has also become an important source of agricultural information, particularly in urban areas, but increasingly in rural regions as well. Television programs dedicated to agriculture often feature experts discussing best farming practices, new agricultural technologies, and success stories of farmers in the region. However, television can be less accessible to rural populations due to its higher costs and the need for infrastructure such as electricity and television sets. For instance, in Ghana, it was important to access information on metrology, new or improved agricultural practice, and agrochemical use [17], and it is used to access information about improved wheat variety [15].

Mobile phone, computer, and combined with the Internet: Mobile phone enable farmers to receive real‐time agricultural information through text messages and calls. It was used to achieve rural development through accessing agricultural information [42]. It is used to remind the farmers about farm management practices to increase productivity [31]. In addition, SMS‐based services are utilized as a means of providing agricultural information to farmers. For example, in Ethiopia, the manager of Ethio Telecom sets up SMS accounts for the target farmers and links them to the phone number of agricultural extension workers. After this, agricultural extension workers send information about new agricultural technologies and climate‐related updates to all the target farmers simultaneously, allowing them to access the latest information and improve agricultural productivity [43]. Computers have also been used to store, analyze, and share agricultural information, making it easier for African farmers to access valuable resources that can enhance their productivity [17].

Internet is still an emerging technology used by farmers to access agricultural information in African countries [23], and 12.5% of the respondents used this as a source of agricultural information [29]. The study done by Van Campenhout et al. [44] in Uganda indicates three types of sources of information on input use and improved maize management practices. These were video, integrating video with interactive voice response, and applying time‐sensitive short message services to deliver information to increase maize productivity.

Moreover, audio–visual materials and Web 2.0 tools were designed to distribute demand‐driven agricultural information for the farmers in Ghana. It includes blogs, instant messaging (IM), Really Simple Syndication (RSS), wikis, and social media. In blogs, the agricultural worker or librarian creates a blog to discuss with the users and get feedback about the information, and it replaces the traditional notice board. IM is important to handle user inquiries during a specific time period. Wiki is a platform used to add or edit content in the system. RSS was aimed at the distribution of information, and it is accessible to the farmers [30].

Extension workers through face‐to‐face contact: In this review paper, extension workers include extension workers and library and information workers, who aim to transfer agricultural information to the farmers and work for the government or nongovernmental organizations. The extension workers working in nongovernmental organizations were also responsible for the transfer of agricultural information to the smallholder farmers [15] and work on accessing information on credit, improved seed varieties, fertilizer type, dosage, and time and method of application [36]. They transfer information to the farmers through demonstration, consultancy service, workshop, and seminar, and health extension workers are also responsible for sharing new agricultural information with women farmers [6].

Colleague farmers, input suppliers, and town criers: include local cooperatives, neighbors, model farmers, farmers′ research groups, and town criers who serve as sources of agricultural information by sharing their experiences. Smallholder farmers access agricultural information from neighbors, friends and model farmers [6]. About 97.40% of the sample respondents access information about the application of fertilizer and weedicides to increase tiger nut (*Cyperus esculentus*) production from colleague farmers [20]. Neighbors and fellow farmers were the sources of information on improved seed/seedling, disease control, and weed control [41]. Farmers′ experience was one of the sources of information through gaining knowledge and practice from their farming experience [37].

Town criers have traditionally been a primary method of disseminating important information, including agricultural advice. They go from village to village, announcing news, updates, and agricultural messages in local language [17]. Input suppliers, such as those providing seeds, fertilizers, and pesticides, are crucial sources of agricultural information. They deliver knowledge to the farmer on the correct use and application of inputs, helping them optimize their yields and minimize losses [6].

Agricultural research centers have played a crucial role in delivering agricultural information to smallholder farmers across African countries by conducting research that directly addresses the challenges of the farmers. These centers focus on developing improved crop varieties, pest management techniques, and sustainable farming practices suited to improve the living conditions of the farmers. They work closely with farmers to test and adapt new technologies, to provide valuable data and recommendations to increase agricultural productivity [6, 15, 26]. In the research center, seed/grain stockists were responsible for providing information on high‐quality, disease‐resistant seed varieties that are suited to local conditions [15].

Printed materials such as posters, magazines, newspapers, school and college notes, books, manuals, and billboards have been essential sources of agricultural information in many African countries, especially in areas with limited access to digital technology or the internet. These printed materials provide a wide range of knowledge, from fundamental agricultural practices to advanced farming techniques [23]. Specifically, school/college notes, books, manuals, and newspapers were the sources of agricultural information in Uganda [26], and libraries were used as the source of information [35].

### 4.2. Distribution of Households by Sources of Agricultural Information

**Figure 2:**
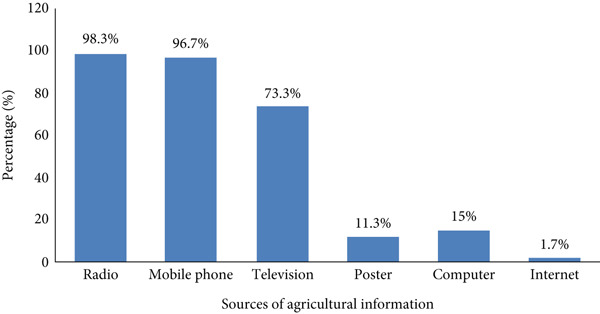
Distribution of respondents by sources of information in Ghana. Source: adopted from Adjoe et al. [17] and Amuda and Thompson [11].

In Ghana, approximately 98.3%, 96.7%, and 73.3% of respondents obtain agricultural information from radio, mobile phone, and television, respectively, showing that these are the primary sources of information in the country. Additionally, around 11.3%, 15%, and 1.7% access information from posters, computers, and internet, respectively (Figure 2).

### 4.3. Constraints of Access to Agricultural Information in Africa

In Africa, access to agricultural information is hindered by challenges associated with farmers, sources of information, the nature of information, extension workers, the distance of farmer training center, and with government (Table 4). The detailed explanation of each constraint is discussed below.

**Table 4:** Constraints of access to agricultural information.

**No.**	**Constraints associated with**	**Types of constraints**
1	Farmers	Illiteracy
		Limited information sharing among farmers
		Religious belief
		Absence of cooperative membership
2	Sources of information	No, unavailability of, and inappropriateness of the aid
		The information is not presented by the aid
		Do not present the issue in local language
3	Agricultural information	Complexity of the information
		High cost of the information and the input
4	Extension workers	Lack of competent and responsible extension workers
		Lack of farmers′ field, training, workshop, and seminar
		Lack of reading materials prepared
		Low rate of extension–farmer linkages
		Poor public relations of the extension agents
5	Distance of the farmer training center	Far distance of the farmer training center from the farmers′ home
6	Government	Lack of rural electrification
		Absence of rural networks

*Note:* literature review, 2025.

#### 4.3.1. Constraints Associated With Farmers

Illiteracy is a major barrier that hinders farmers′ ability to seek information, to effectively use available aid, and to search on where the information is. Additionally, illiterate households did not read the printed materials, so they are not willing to access the agricultural information from printed materials ([14, 41]; Manteaw, 2022). Limited information sharing also restricts access to agricultural information in Africa by creating barriers to the flow of knowledge among farmers. When farmers are not inclined to share their experiences, techniques, or insights with one another, it reduces opportunities for learning and collaboration. Without a culture of information sharing, farmers may miss out valuable advice or updates about new farming practices, pest control methods, market trends, or government policies [26].

Religious beliefs can restrict access to agricultural information in Africa by influencing the acceptance of certain farming practices, technologies, or advice. In some cases, traditional or religious views may conflict with modern agricultural methods, such as the use of genetically modified crops, chemical fertilizers, or advanced irrigation techniques. For example, some religious groups may view these technologies as unnatural or against their spiritual beliefs, making farmers hesitant to embrace them, even if they could improve productivity, and the absence of cooperative membership restricts access to agricultural information in Africa by limiting farmers′ ability to share and access agricultural information among group members. Hence, cooperatives often serve as a vital platform for distributing relevant agricultural information used to improve the agriculture sector [10].

#### 4.3.2. Constraints Associated With Sources of Information

Farmers might not have radio and television to access agricultural information [10]. In addition to this, when aid is unavailable, farmers miss out on critical information to achieve agricultural productivity. If the aid provided is inappropriate for the specific needs or context of the farmers, it becomes ineffective. For example, if the aid consists of information or technologies that do not align with local farming practices, environmental conditions, or cultural preferences, farmers may find it difficult to implement or trust it [26, 45]. Furthermore, the absence of agricultural information on radio or television prevents farmers in Africa from accessing essential knowledge. Another factor is the language of instruction; when programs are conducted by english rather than local language, it restricts farmers′ access to essential agricultural information; hence, the farmers did not understand the language [10, 37].

#### 4.3.3. Constraints Associated With Agricultural Information

The complexity of the information, the high cost of information, and input influenced access to agricultural information by the farmers. Agricultural information often involves technical knowledge, which can include aspects such as advanced farming techniques, pest management, crop rotation, soil health, and climate adaptation. The complexity of the information can create barrier to fully understand the issues, and this discourages the households to access the information in African countries [10]. Additionally, the high costs of information and inputs limit farmers′ access to agricultural knowledge, discouraging them from adopting new technologies that could benefit them in the future [37, 45].

#### 4.3.4. Constraints Associated With Extension Workers

Extension workers play a crucial role in disseminating agricultural knowledge and helping farmers adopt best practices. However, many regions in Africa suffer from a shortage of well‐trained, competent, and responsible extension workers to share new information and practices to the farmers. In most African countries, extension workers did not implement farmers′ field, training, workshops, and seminars. This restricts the farmers′ access to new agricultural information and technology [20], and they did not prepare reading materials and store them in the farmer training center. This influences the households to access information [14, 46].

In addition to these, the low rate of extension–farmer linkages and poor public relations of the extension workers significantly hinder access to agricultural information in Africa. Without strong, consistent communication between extension workers and farmers, it becomes difficult to effectively deliver knowledge, foster trust, and encourage the adoption of new practices [14]. Similarly, poor public relations prevents farmers from fully engaging with extension workers, further limiting the farmers to access valuable information [20].

Despite these, the absence of extension workers, who are essential to provide guidance, training, and expert advice, means that farmers are left without personalized, localized support tailored to their specific needs. Without extension workers who can directly engage with farmers through farm visits, workshops, or local training programs, the dissemination of agricultural information is stunted, leaving farmers with inadequate knowledge on best practices, new technologies, and effective solutions to challenges such as pests, diseases, and climate change [10].

#### 4.3.5. Constraints Associated With the Distance of the Farmer Training Center

The far distance of farmer training center from farmers′ home was a major challenge of the farmers to access crucial agricultural information in many African countries, especially for those in remote and rural areas. Farmers often face significant travel costs, time constraints, and physical barriers, such as poor road infrastructure and a lack of transportation options, which discourage them from attending training sessions, workshops, and seminars. As a result, they miss valuable opportunities to learn about new agricultural technologies, improved farming practices, pest management strategies, and market trends that could enhance their productivity and livelihoods [14].

#### 4.3.6. Constraints Associated With the Government

Lack of rural electrification and absence of rural network influenced the farmers to access agricultural information in most African countries. The absence of rural electrification exacerbates the difficulty of powering on computers, mobile phones, radio, and television and this hinders the farmers to access agricultural information about weather patterns, market prices, new farming techniques, or pest control methods from these aids [10, 14, 47]. Moreover, the absence of rural communication network, such as internet connectivity or mobile network, further isolates farmers from the wealth of online agricultural resources and mobile‐based advisory services. This lack of connectivity prevents farmers from engaging in real‐time interactions with extension workers, agricultural experts, or other farmers, severely limiting their access to crucial information [6, 12, 14, 26].

## 5. Conclusion and Recommendation

### 5.1. Conclusion

Agriculture is the main livelihood option of farmers in African countries. Agricultural information on new practices and technology is important to increase agricultural productivity. This review indicates that radio, television, mobile phone, computer with Internet, face‐to‐face contact by extension workers, colleague farmers, input suppliers, town criers, agricultural research centers, and print materials, including posters, magazines, newspapers, school/college notes and books, manuals, billboards, and bulletins, were the sources of agricultural information in Africa. Illiteracy; limited information sharing among farmers; religious beliefs; lack of cooperative membership; the unavailability or inappropriateness of information sources; absence of aids to present the information; the failure to use local language; complex information; high cost of both information and inputs; inadequate or unqualified extension workers; a lack of farmer training, workshops, and seminars; insufficient reading materials; low extension–farmer linkages; poor public relations by extension workers; the long distance of training centers from farmers′ home; lack of rural electrification; and the absence of rural network were constraints which influnce the farmers to access agricultural information. Increasing agricultural productivity by utilizing new information and technology was important to reduce poverty and food insecurity in Africa.

### 5.2. Recommendation

➢ There should be coordination of governmental and non‐governmental organizations to transfer of new agricultural technology to the farmers.
➢ The government should focus on the expansion of rural road infrastructure to facilitate the diffusion of information to the farmers.
➢ The Agricultural extension worker should be committed to providing agricultural information to the farmers.
➢ There should be a strong research–extension–farmer linkage to generate demand‐driven agricultural technology for the farming communities.

## Ethics Statement

The author has nothing to report.

## Disclosure

The author approves the full manuscript.

## Conflicts of Interest

The author declares no conflicts of interest. Melese Abebaw Abate is currently a lecturer and researcher in the Department of Rural Development and Agricultural Extension, Mekdela Amba University, Ethiopia. His research interest includes agricultural information, livelihood, and food security.

## Author Contributions

Melese Abebaw Abate: conceptualization, literature search, organization of information, interpretation, drafting, and writing the full manuscript.

## Funding

No funding was received for this manuscript.

## Data Availability

Data and material can be made available.
